# A retrospective study of nonneoplastic and neoplastic disorders of the salivary glands

**DOI:** 10.1097/MD.0000000000035751

**Published:** 2023-10-20

**Authors:** Sorin Vamesu, Oana Andreea Ursica, Ana Maria Gurita, Raluca Ioana Voda, Mariana Deacu, Mariana Aschie, Madalina Bosoteanu, Georgeta Camelia Cozaru, Anca Florentina Mitroi, Cristian Ionut Orasanu

**Affiliations:** a Clinical Service of Anatomic Pathology, Departments of Pathology, “Sf. Apostol Andrei” Emergency County Hospital, Constanta, Romania; b Faculty of Medicine, “Ovidius” University of Constanta, Constanta, Romania; c Center for Research and Development of the Morphological and Genetic Studies of Malignant Pathology (CEDMOG), “Ovidius” University of Constanta, Constanta, Romania; d Academy of Medical Sciences of Romania, Bucharest, Romania.

**Keywords:** neoplastic salivary gland, nonneoplastic salivary gland, parotid, pleomorphic adenoma, Warthin tumor

## Abstract

The spectrum of major and minor salivary gland disorders varies widely. Epidemiological data on some injury categories are rare and often not up-to-date. This study aims to analyze epidemiological data using clinical, paraclinical, and histopathological parameters. Study was carried out for 5 years on the nonneoplastic and tumoral pathology of the salivary glands. Data were statistically analyzed using the appropriate parameters. Data analysis according to the biological behavior of the lesions revealed great heterogeneity. Statistically significant correlations were observed between the type of injury, age (*P* = .002) and gender (0.033). The environment of origin of the patients as well as the comorbidities reflected in most cases the nature of the process. Associations were also observed between the biological behavior of the lesions and the hemicranial topography (*P* = .019), the type of salivary gland (*P* = .024), and the surgical technique used (*P* < .001). Most cases were identified in the major salivary glands, often in the parotid. The most common diseases are represented by nonspecific chronic sialadenitis (nonneoplastic lesion), pleomorphic adenoma and Warthin tumor (benign tumors), mucoepidermoid carcinoma (malignant tumor), and squamous carcinoma (secondary tumor). They presented axial diameters between 2 to 95 mm. The most used curative technique was subtotal excision with facial nerve preservation. In conclusion, the study highlighted the main epidemiological aspects of salivary gland disorders. Some data agree with the specialty literature, and particular aspects are also observed. Therefore, this research is useful both in the medical and research fields.

## 1. Introduction

**Figure 1.**
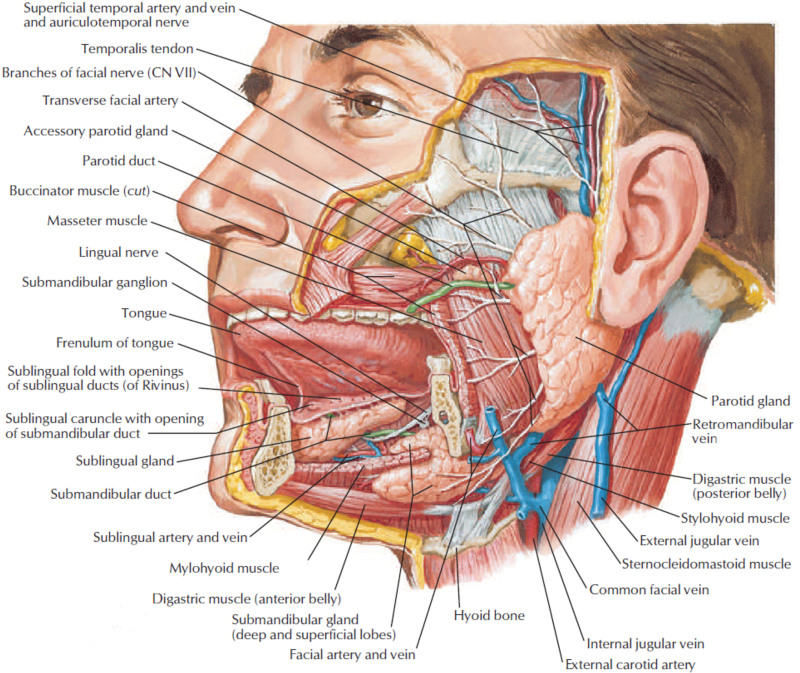
The anatomical region of the major salivary glands.^[1]^

The salivary glands are located in the upper aerodigestive tract (oral cavity and oropharynx). They are represented by minor salivary glands and major salivary glands: parotid, submandibular, and sublingual (Fig. 1). These are exocrine organs whose main function is the secretion of saliva.^[1–3]^

The spectrum of pathological lesions identified at their level is broad and diverse. These lesions can be developmental, functional, inflammatory, infectious, iatrogenic, obstructive, traumatic, autoimmune, degenerative, vascular, or neoplastic disorders (Table 1).^[4,5]^ Except for the last element listed above, the lesions can be grouped under the term nonneoplastic diseases of the salivary glands. Data about their incidence are very rare to find. Until now, it is only known that they have a maximum incidence between the second and 4th decades of life, predominantly affect the female gender, and are frequently located at the level of the major salivary glands.^[6,7]^

**Table 1:** The main diseases of the salivary glands.^[4]^

Salivary gland disorders	Main causes
Developmental	Abberancy, Aplasia, Atresia
Functional	Sialorrhea, Xerostomia
Inflammatory	Chronic sclerosing sialadenitis, Post irradiation sialadenitis
Infectious	Acute bacterial infection, Chronic bacterial infection, Mumps, Viral infection
Obstructive	Mucocele, Mucose retention cyst, Ranula
Traumatic	Sialolithiasis
Autoimmune	Mikulicz disease, Sjogrens syndrome
Degenerative	Idiopathic sialolithiasis
Vascular	Necrotizing sialometaplasis
Neoplastic	Acinic cell carcinoma, Adenoid cystic carcinoma, Basal cell adenoma, clear cell carcinoma, Lymphomatosum, Pleomorphic Adenoma, Mucoepidermoid Carcinoma, Oncocytoma, Papillary Cystadenoma

Neoplastic lesions represent approximately 5% of head and neck tumors worldwide, with a slightly increased frequency in Europe (8.5%).^[7,8]^ They have an incidence of <5 per 100,000 inhabitants.^[9]^ More than 70% of tumors are of epithelial origin. They are often identified in the parotid, less often in the submandibular (10–25%), minor salivary glands (9–20%), and sublingual (1–5%).^[10]^ About 80% of these are benign, with an increased frequency in the major salivary glands (−70%).^[9,11]^ Malignant tumors are very rare, with an incidence of <1.3 per 100,000 inhabitants. They occur frequently in males and in the 4th and fifth decades of life.^[7,9]^ In half of the cases, they are located at the level of minor salivary glands. When they are found in the major salivary glands, the submandibular and sublingual glands are most frequently affected.^[9,11]^ Secondary neoplastic lesions are almost exclusively at the level of the parotid and submandibular gland. Most commonly, they originate from a squamous cell carcinoma or a melanoma.^[12]^

In addition to clinical aspects, the correct diagnosis must consider imaging and histopathological features.^[13]^ From a clinical point of view, the lesions can be asymptomatic (−40%) or they can often be manifested by swelling, pain, and fever, with a sudden or insidious onset. It should be noted that any painless swelling, without inflammatory signs, should raise suspicions of malignancy.^[14,15]^

The topography, dimensions, extension, number, and main architectural features can be evaluated through imaging examinations. The first line is represented by ultrasound. This identifies the intraglandular belonging and the type of lesion (cystic or solid).^[16]^ In cases of deep localization, the use of computer tomography or magnetic resonance imaging is recommended. For example, given the increased risk of malignant lesions at this level, in lesions of the sublingual gland, magnetic resonance is imperatively recommended.^[17]^

Current epidemiological data covering all aspects of salivary gland lesions are very rare. In addition, the great heterogeneity of both nonneoplastic and neoplastic lesions requires a complex analysis that combines data on clinical and histopathological aspects. Therefore, the purpose of this study is to make a current report on the salivary gland pathology of both neoplastic and nonneoplastic entities. Furthermore, another objective of this study is to conduct a concise literature review, focusing on the latest information regarding these conditions, with the aim of improving patient management strategies.

## 2. Material and method

We conducted a retrospective study for a period of 5 years (2018–2022) of patients diagnosed with salivary gland pathology hospitalized at the Constanta County Emergency Clinical Hospital, Dobrogea. The data were extracted from the hospital’s archives and electronic databases. The inclusion criteria consist of adult patients with nonneoplastic and neoplastic lesions. Recurrences, patients under the age of 18, and cases diagnosed by autopsy represent exclusion criteria.

The clinical and paraclinical information of the patients came from the hospitalization form. The imaging examinations were either performed in the ambulatory or the hospital. For those who underwent an imaging examination in the hospital, data could be collected regarding the topography, dimensions, extension, presence of adenopathies, and the maximum diameter of the lymph nodes.

Sampled tissues were firstly, macroscopically described and then prepared according to international protocols, up-to the stage of microscopic slides in Hematoxylin-Eosin staining within the Clinical Anatomical Pathology Service of Constanta. The histopathological diagnosis was given by 2 pathologists in accordance with the latest international criteria, as well as with the latest World Health Organization classification (5th edition, 2022).

Statistical data analysis was performed in SPSS Statistics Version 26 (IBM Corporation, NY). Indicators of central tendency and variability were used. The categorical data was analyzed through the chi-squared test and Fisher exact test, while the continuous variables were examined using the Mann–Whitney *U* test and Kruskal–Wallis H test. To establish the association of data, we used the Pearson correlation coefficient. Results were considered statistically significant at a value of *P* < .05.

The study was conducted in accordance with the Declaration of Helsinki and approved by the Institutional Ethics Committee of Constanta County Emergency Hospital (No. 04/April 06,2023).

## 3. Results

**Figure 2.**
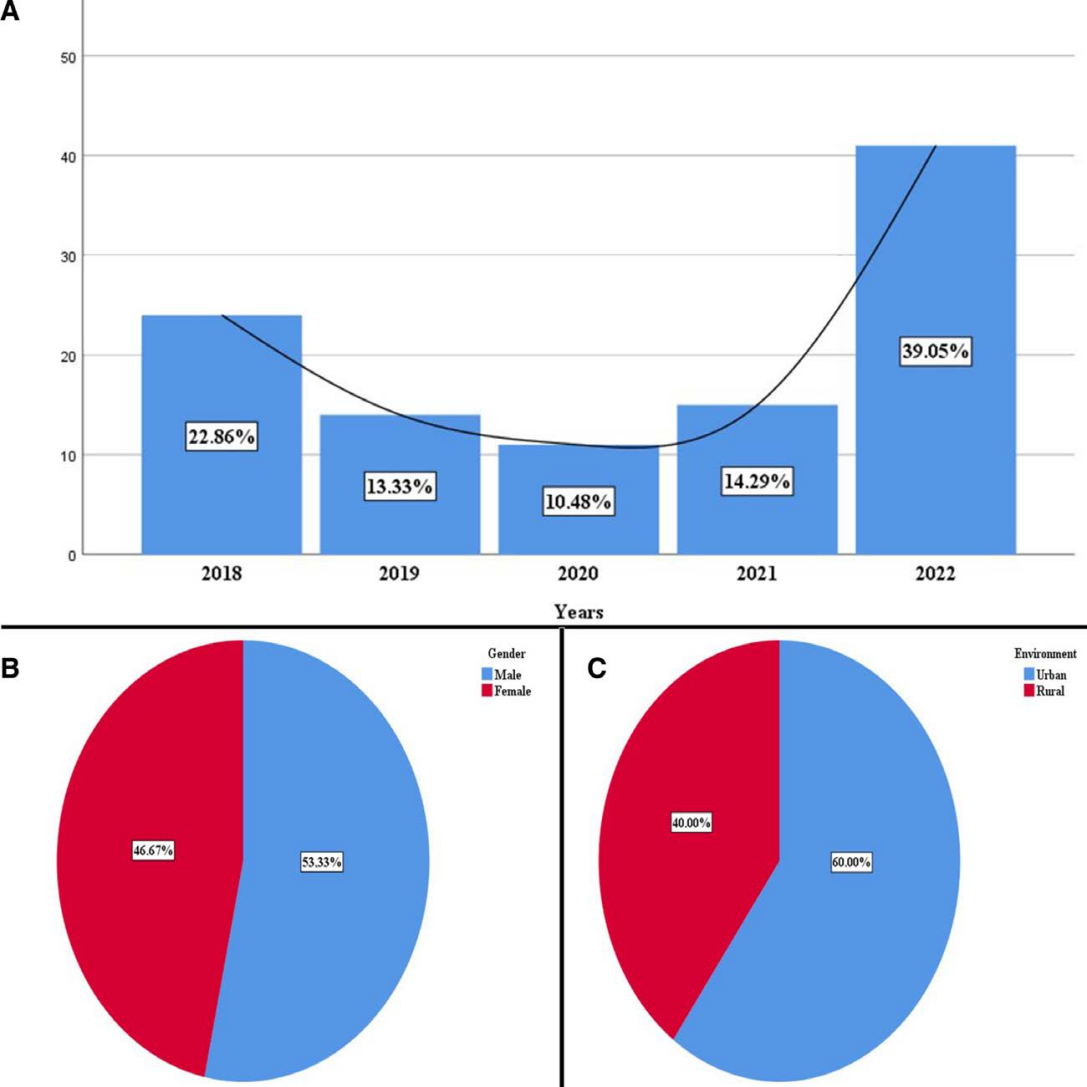
(A) Distribution of cases by years of study. (B) Distribution of cases by gender. (C) Distribution of cases by the environment.

The retrospective analysis identified 105 cases of salivary gland lesions. Their distribution was undulating with 2 peaks in 2018 (22.86%) and 2022 (39.05%) (Fig. 2A). At the time of diagnosis, the average age was 56.59 years (20–91 years), with increased frequency in the 7th decade of life (25.71%). The most affected was the male gender 53.33% (Fig. 2B). The majority of patients (60%) originate from urban areas (Fig. 2C).

Among the patient’s comorbidities, the most common were hypertension (31.43%), diabetes (29.52%), and obesity (6.67%). The rare cases when other chronic diseases were present (9.52%) such as viral or ethanol hepatitis, Sicca syndrome, or hypothyroidism should not be neglected. A minority of patients (13.33%) presented malignant pathology (squamous carcinoma, melanoma, hepatocarcinoma, or breast carcinoma), some cases showing extension to the salivary glands.

The most frequent biochemical abnormalities encountered were anemia (3.81%), acute inflammatory syndrome (2.86%), and thrombopenia (2.86%). More than half of the cases (54.29%) benefited from an imaging examination. The rest of the patients came with the examination from the ambulatory. An increased frequency of only jugular (41.86%), submandibular and jugular (23.26%), and laterocervical (16.28%) adenopathies were identified. Their average maximum diameter was 13.63 mm (6–26 mm).

After the stratification according to the biological potential, the following data regarding the socio-demographic distribution in Table 2 could be observed.

**Table 2:** Demographic distribution of the cases divided by biological behavior.

	Nonneoplastic lesions (n = 20)	Benign tumor lesions (n = 65)	Malignant tumor lesions (n = 12)	Secondary tumor lesions (n = 8)	*P* value
Age (yr)	54.40 (32–81)	53.88 (20–88)	68.17 (33–78)	66.75 (50–91)	.002
Decade (average)	6 (35%)	7 (29.23%)	8 (66.67%)	6 (37.50%)	.023
Gender					
Female	50%	52.31%	41.67%	0%	.033
Male	50%	47.69%	58.33%	100%	
Environment					
Urban	45%	64.62%	66.67%	50%	.380
Rural	55%	35.38%	33.33%	50%	
Comorbidities					
Hypertension	10%	35.38%	41.67%	37.50%	.107
Diabetes	10%	32.31%	50%	25%	.079
Obesity	0%	9.23%	0%	12.50%	.326
Adenopathy					
Jugular	50%	34.78%	33.33%	75%	.653
Submandibular	0%	26.09%	0%	0%	
Laterocervical	20%	13.04%	33.33%	0%	
Submandibular and jugular	30%	17.39%	33.33%	25%	
Laterocervical and jugular	0%	8.70%	0%	0%	
The maximum diameter of the adenopathies (mm)	14.70 (10–26)	12 (6–15)	14.50 (11–20)	19 (16–26)	.081

An overwhelming majority of the lesions were identified in the major salivary glands (89.52%), the predilection location being the parotid (77.14%). Most diseases were observed at the level of left hemicranium (49.52%). The most used surgical excision technique was subtotal resection with preservation of nerve structures (45.71%). After finalizing the diagnosis, a complete resection was observed in 72.38% of cases.

According to biological potential, we noticed that most cases were benign tumor lesions (61.90%), followed by nonneoplastic diseases (19.05%) and malignant tumor lesions (11.43%). One-third of them were accompanied by inflammatory processes: chronic (17.14%), active chronic (15.24%), or active (0.95%). The intensity was mostly moderate (45.71%). Also, in 13.33% and 5.71% of the cases, the phenomena of hemorrhage and necrosis were identified, respectively. Excised lymph nodes were identified in only 37.14% of the cases, the majority (92.31%) having a reactive character.

**Figure 3.**
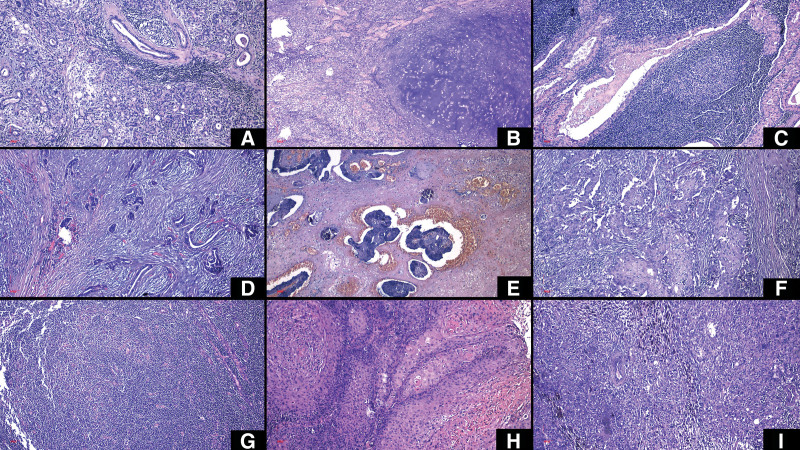
Collage of microscopic images of the most relevant cases, in usual Hematoxylin-Eosin staining, objective x100. (A) Nonspecific chronic sialadenitis. (B) Pleomorphic adenoma. (C) Warthin tumor. (D) Polymorphous adenocarcinoma. (E) Acinic cell carcinoma. (F) Salivary duct carcinoma. (G) Non-Hodgkin lymphoma. (H). Squamous cell carcinoma metastasis. (I) Malignant melanoma metastasis.

The most common pathologies identified were chronic nonspecific sialadenitis (35% of nonneoplastic cases), pleomorphic adenoma and Warthin tumor (47.69%, respectively 43.08% of cases of benign tumor lesions), mucoepidermoid carcinoma (25% of cases of malignant tumor lesions) and squamous carcinoma (25% of cases of secondary tumor lesions) (Fig. 3) (Table 3). Lymphovascular and perineural invasions were observed in similar percentages in malignant lesions (40%).

**Table 3:** The distribution of the pathology in the study group.

	Diagnosis	Frequency in the category (total)	M:F* ratio	Average age	Salivary gland	
Nonneoplastic	Nonspecific chronic sialadenitis	35% (6.67%)	1:0.75	57.86	Accessory	28.57%
	Normal appearance	25% (4.76%)	0.66:1	58	Parotid	3.70%
	Chronic nonspecific sialadenitis with active character	15% (2.86%)	0.5:1	41.33	Parotid	2.47%
	Subacute sialadenitis	10% (1.9%)	2:0	62	Sublingual	20%
	Branchial cleft	5% (0.95%)	1:0	62	Parotid	1.23%
	Sarcoidosis	5% (0.95%)	0:1	36	Parotid	1.23%
	Mucocele	5% (0.95%)	0:1	47	Lip	50%
Benign tumor	Pleomorphic adenoma	47.69% (29.52%)	0.35:1	44.42	Parotid	30.86%
	Warthin tumor	43.08% (26.67%)	2.5:1	62.96	Parotid	34.57%
	Myoepithelioma	4.62% (2.86%)	1:0.5	65	Parotid	3.70%
	Basal cell adenoma	1.54% (0.95%)	0:1	46	Parotid	1.23%
	Lipoma	1.54% (0.95%)	1:0	43	Parotid	1.23%
	Intercalated duct adenoma	1.54% (0.95%)	0:1	78	Parotid	1.23%
Malignant tumor	Mucoepidermoid carcinoma	25% (2.86%)	3:0	69.67	Parotid	3.70%
	Polymorphous adenocarcinoma	16.67% (1.90%)	1:1	64.50	Lip	50%
	Histiocytosis X	8.33% (0.95%)	1:0	77	Submandibular	12.50%
	Non-Hodgkin lymphoma with small B cell	8.33% (0.95%)	1:0	33	Parotid	1.23%
	Acinic cell carcinoma	8.33% (0.95%)	0:1	78	Submandibular	12.50%
	Epithelial-myoepithelial carcinoma	8.33% (0.95%)	0:1	77	Parotid	1.23%
	Salivary duct carcinoma	8.33% (0.95%)	1:0	78	Parotid	1.23%
	Adenocarcinoma NOS†	8.33% (0.95%)	0:1	73	Palatine vault	50%
	Plasmacytoma	8.33% (0.95%)	0:1	64	Parotid	1.23%
Secondary tumor	Squamous cell carcinoma	50% (3.81%)	4:0	59	Accessory	28.57%
	Melanoma	12.50% (0.95%)	1:0	81	Parotid	1.23%
	Baso-squamous carcinoma	12.50% (0.95%)	1:0	54	Parotid	1.23%
	Trichilemmal carcinoma	12.50% (0.95%)	1:0	91	Parotid	1.23%
	Infiltrative basal cell carcinoma	12.50% (0.95%)	1:0	72	Parotid	1.23%

NOS = not otherwise specified.

* Male: Female.

† Not otherwise specified.

Stratification according to biological potential revealed the following data presented in Table 4.

**Table 4:** Distribution of cases from the study group divided by biological character according to histo-anatomical aspects.

	Nonneoplastic lesions (n = 20)	Benign tumor lesions (n = 65)	Malignant tumor lesions (n = 12)	Secondary tumor lesions (n = 8)	*P* value
Topography					
Right	50%	44.62%	75%	37.50%	.019
Left	50%	55.38%	25%	37.50%	
Central	0%	0%	0%	25%	
Salivary gland					
Major	85%	95.38%	75%	75%	.024
Minor	15%	4.62%	25%	25%	
Anatomical glandular structure					
Parotid	50%	90.77%	58.33%	62.50%	<.001
Submandibular	20%	3.08%	16.67%	0%	
Sublingual	15%	1.54%	0%	12.50%	
Accessory	15%	4.62%	25%	25%	
The maximum diameter of the lesions (mm)	23.65(2–79)	32.86(10–95)	26.75(9–75)	35.38(6–94)	.077
Surgical approach					
Biopsy	45%	1.54%	25%	50%	<.001
Enucleation	5%	16.92%	25%	0%	
Total excision	35%	18.46%	41.67%	12.50%	
Subtotal excision	15%	63.08%	8.33%	37.50%	

A significant correlation was found among older adults, where the localization of lesions was most prevalent on the right side of the head, followed by the central region and then the left side (*P* = .015). Also, we noticed that advanced age was associated with an incomplete excision (*P* = .007).

We noticed that there is a difference in the distribution of lesions depending on the age and gender of the patients. Thus, an older mean age was observed in men (60.68 years vs 51.91 years in women) (*P* = .005).

Our findings revealed a significant correlation between benign and nonneoplastic lesions with the female gender. However, a higher likelihood of malignant and secondary lesions was observed in the male gender (*P* = .033). Also, the amplitude of the intralesional inflammatory process was higher in females (*P* = .013).

Malignant tumors were associated with larger lymph node diameters than nonneoplastic lesions followed by benign tumors (*P* = .005). Also, an increased average of the maximum diameter was found in the cases of patients with thrombocytopenia and anemia (*P* = .043).

Benign tumoral lesions were associated with an increased presence in the major salivary glands, while the other types of lesions had a predominance in the minor salivary glands (*P* = .024). Among the types of salivary glands, a correlation of the parotid with benign lesions, of the submandibular gland with malignant and nonneoplastic lesions, and of the sublingual and accessory glands with secondary and nonneoplastic lesions was observed (*P* < .001).

Larger lesions were associated with localization in the major salivary glands (*P* < .001). More specifically, the largest lesions were observed in the parotid gland, followed by the submandibular and sublingual glands (*P* = .007).

The size of the lesions was also associated with the type of surgical intervention. In large lesions, subtotal excision was preferred, followed by total excision and enucleation (*P* < .001). Also, the surgical technique showed statistically significant associations depending on the lesion type. Thus, benign tumors were associated with subtotal excision and enucleation, nonneoplastic lesions with biopsy and complete excision, secondary tumors with biopsy, and malignant tumors with biopsy and subtotal excision (*P* < .001). Corroboration of these data with the histopathological evaluation of the excision revealed significant statistical associations in the case of complete excision only with benign tumors. The rest of the lesions were associated with incomplete excisions (*P* < .001). The presence of intralesional necrosis was associated with an incomplete excision (*P* = .048). Except for the nonneoplastic lesions, the inflammatory process, especially the lymphoplasmacytic one, was associated with malignant and secondary tumor lesions (*P* < .001).

## 4. Discussions

The present epidemiological study on salivary gland lesions in the adult population represents an analysis that can be used in the prognosis and planning of medical services, representing a basis both for future research and for good patient management.

Over a period of 5 years, 105 cases were identified in patients aged between 20 and 91 years (56.59 years). We observed a younger age distribution for benign tumors and nonneoplastic lesions and an older age for malignant tumors and metastases. The mean age as well as the age distribution by pathology were similar to the studies carried out by Aldeaimi AA et al and Zurek M et al^[8,13]^

The distribution according to the patient’s gender is in accordance with the literature, observing a slightly increased frequency in males.^[18–20]^ The predominance of tumor lesions on female gender is known (aspect also identified by us).^[11,21]^ In the present study, we noticed that malignant lesions were identified more frequently in the male gender. The literature on the association between gender and nonneoplastic lesions is insufficient. Some studies suggest that women are more affected, but the current study finds an equal number of cases among both genders.^[4,13]^

Depending on the environment of origin of the patients, a predominance of tumor lesions was noted in the case of the urban environment. nonneoplastic lesions, especially inflammatory ones, developed in those of rural origin. Recent research on surgical excision specimens has highlighted a predominance of neoplastic lesions in rural areas as well.^[22,23]^

This aspect can be attributed to people’s occupations. A case-control study carried out in France noted a strong association of the occupational factor in the development of malignant lesions of the salivary glands. The most prone occupations were represented by agricultural workers (farmers, animal breeders).^[24]^ In contrast, studies that combined cytodiagnosis obtained through fine needle aspiration found a higher occurrence of nonneoplastic lesions, particularly inflammatory ones, in rural areas.^[25–27]^

Besides the occupational factor, a series of other events can lead to salivary gland injuries. For nonneoplastic lesions, the most common causes are represented by Staphylococcus Aureus, anaerobic germs, human immunodeficiency virus, influenza, or Sjogren syndrome.^[28,29]^ Associations with the Epstein-Barr virus or human immunodeficiency virus have been observed for benign tumoral lesions.^[30]^ In the malignant tumors, the most incriminated factor is human papillomavirus infection, the most common phenotypes being 16 or 18.^[30,31]^ Added to these risk factors are patient comorbidities such as alcohol consumption, tobacco, and obesity, but these do not show statistically significant associations.^[32,33]^ In this study group, it was not possible to determine the consumption of alcohol and tobacco. Instead, the most common comorbidities are hypertension, diabetes, and obesity. Their frequency registers high rates in tumor lesions, especially in malignant ones. The first step in the preoperative diagnosis of salivary gland lesions is based on imaging and cytological methods.^[34]^ The most used technique is represented by ultrasound. The method offers several benefits, such as being noninvasive, cost-effective, providing precise localization of superficial lesions (particularly in the parotid region), and rapid identification of acute inflammatory conditions caused by sialolithiasis.^[34,35]^ In nonneoplastic lesions, the ultrasound examination highlights hypoechoic signals (caused by edema), gland enlargement with a diffuse decrease in echogenicity (acute inflammation), gland enlargement with heterogeneity (Sjorgen syndrome) or reduced gland with calcifications (chronic inflammation).^[35,36]^ Similar aspects can be highlighted in benign lesions. In these cases, a superior imaging technique or cytological examination by fine needle aspiration is recommended.^[34,35]^ Magnetic resonance imaging or computed tomography are recommended for detecting lesions in minor and sublingual glands, as well as for distinguishing between benign, malignant and secondary tumors.^[34]^ The aspects that argue for a malignant lesion over a benign 1 consist of capsule rupture, invasion of adjacent structures, poorly defined edges, or perineural extension.^[16,35,36]^ Computed tomography can provide additional data regarding the presence of calcifications, lithiasis, or the formation of gas bubbles.^[35]^ In the secondary lesions, most frequently originating from melanoma or a squamous carcinoma (as was also found in the present study), magnetic resonance imaging highlights multifocal lesions, with poorly defined edges with perineural extension.^[16]^ When imaging cannot specify the nature of the lesion, fine needle aspiration is indicated.^[17]^

Accurate evaluation of lymph node status in the head and neck region is crucial for both malignant tumors (for proper staging and guidance of radiotherapy) and benign or nonneoplastic conditions. Imaging, can be appreciated by the nodular shape if it is reactive (oval shape) or invaded (round shape). When evaluating lymph nodes, besides their shape, it’s important to consider their maximum diameter. For the laterocervical and jugular lymph nodes, a diameter of 10 to 11 mm is the reference value for reactive or neoplastic changes. If examining retropharyngeal lymph nodes, a diameter of 5 mm is considered.^[37]^ This quantification of the diameter was also observed in the present study. Lymph node quantification is suggested as a supplementary staging technique in conjunction with TNM, as it appears to have a significant effect on survival, especially in cases of malignant salivary gland tumors.^[38,39]^

The preferential site of tumor lesions is the parotid, less often in the other structures.^[34,40]^ This aspect was also noted in the present study, the benign tumor pathology was located in an overwhelming majority in the parotid (90.77% of cases). The sublingual location raises great signs of concern; at this level almost exclusively malignant lesions are identified. In our case, no primary malignant lesions were registered at the sublingual level, but only secondary ones.^[34,41]^ The accessory salivary glands are a rare location; the most frequent lesions are observed at the level of the palate.^[42,43]^ In primary and secondary malignant tumor pathology, the most common lesions were found to affect the floor of the mouth and the minor glands of the lip.

An aspect not found in the specialized literature was the topographical location of the lesions and their biological potential. We have found a significant correlation between the occurrence of malignant lesions on the right side of the hemicranium, benign and nonneoplastic lesions on the left side, and secondary lesions in the middle of the hemicranium (*P* = .019). Regarding the most common types of lesions, the frequency identified by us is in some places similar to international research. This aspect supports the idea of heterogeneity of salivary gland pathology. nonneoplastic pathology can be found under different classifications: etiological or depending on the presence or absence of inflammation.^[6]^ However, the most common entities encountered are represented by inflammation (especially chronic nonspecific sialadenitis) and cystic lesions (mucocele).^[44]^ Benign tumor pathology has a higher incidence. The most frequent lesions are represented by pleomorphic adenoma, followed at a short distance by Warthin tumor.^[10,20,45]^

The prevalent types of primary malignant pathology are adenoid cystic carcinoma and mucoepidermoid carcinoma, as stated by different authors in literature.^[4,10,20,45,46]^ Contrary to other studies where patients were found to be in advanced stages (pT4 or pT3), a noteworthy 71.43% of the patients in our present study, who underwent surgery rather than just a biopsy, were discovered to be in the early stages (pT1 and pT2).^[45,47,48]^ Another element of major importance in the case of these tumors is represented by perineural and/or lymphovascular invasion. An American study, conducted on a group of 137 patients with malignant pathology, noted an increased frequency of cases of perineural invasion (67.9%) and lymphovascular invasion (42.3%).^[48]^ In our group of patients, their frequency was lower (40% in each case). This aspect can be explained either by the advanced stage identified by them (pT3 or pT4), or by the fact that it has been observed that certain cancers (carcinoma ex pleomorphic adenoma, salivary duct carcinoma, adenocarcinoma, acinic cell carcinoma) have a higher risk of spreading, features rarely or not at all found in our batch.^[48]^

The most common secondary malignant pathologies of the salivary glands can reach predominantly by the lymphatic route - as in the case of the parotid - or hematogenous - in the case of the submandibular glands. Most parotid metastases come from primary cancers of the head and neck skin and less often from other sources.^[12,49]^ This aspect was also highlighted in the present study. Skin cancers (not only squamous cell carcinoma) occurred mostly at the supraclavicular level, most commonly spreading via the neural pathway. Melanoma has the second-highest frequency in cases of metastases in the salivary glands, especially in the parotid.^[50,51]^

In the histopathological evaluation, a major element of neoplastic lesions begins to derive from the presence of the inflammatory infiltrate. In this study, the presence of a rich lymphoplasmacytic inflammatory infiltrate was associated with malignant lesions. There are future perspectives on the exploitation of this lymphocyte tumor infiltrate for the application of immunotherapies.^[52]^ Currently, the prognostic and predictive aspect of the tumor microclimate is being studied to apply therapies with Programmed cell death ligand 1 inhibitors.^[52,53]^ In the case of benign tumors, inflammation appears to play a crucial role in the development of tumors and can continue to exist throughout the growth process due to its ability to promote cell proliferation.^[54]^

The mean maximum diameter of the tumors supports the heterogeneity of this pathology. In the present study, we observed that the largest lesions were secondary malignant ones, followed by benign tumors. A paraclinical study by Matsuda E et al^[55]^ identified larger sizes in the cases of primary malignant lesions, compared to benign ones. These aspects denote the fact that tumoral lesions do not follow a pattern, and the infiltrative nature given by the malignant character does not always materialize in increased sizes. The importance of the dimensions is a special 1. Based on these, the TNM classification of malignant tumors is carried out, as well as the guidance of the surgical approach technique.^[56,57]^ In the present study, we observed an association of the surgical technique depending on the maximum diameter of the lesion.

Surgical treatment is recommended whenever a tumor lesion is suspected.^[14,15,58]^ The differential diagnosis between a benign pathology and a malignant 1 is difficult from a clinical point of view, sometimes even paraclinical. It can be presumptively established by biopsy.^[14]^

In the cases of parotid tumor lesions, the surgical technique is varied depending on the biological nature of the process. The most common technique is superficial parotidectomy with dissection of the facial nerve. Other techniques are represented by: superficial partial parotidectomy (it may be sufficient for benign cases); enucleation which presents the risk of facial nerve damage or recurrence; excision with preservation of the facial nerve in small lesions; total parotidectomy or radical or extended radical parotidectomy is preferred in malignant cases.^[15,59,60]^

In submandibular locations where the tumor is small, low-grade, or well-defined, enucleation can be performed. Otherwise, the adjacent lymph nodes are also excised bilaterally. In the sublingual location, the technique includes wide surgical excision. In case of lymphadenopathy, dissection of the lymph nodes will be performed.^[15]^

For minor or accessory salivary gland tumors, there are 2 main surgical approaches. First, for benign lesions, tumor excision alone is performed. Second, for malignant lesions, tumor excision is combined with surgical margins and lymph node dissection. In malignant cases, adjuvant irradiation can be performed (large lesions - usually over 4 cm, high grade, incomplete resection margins, or adenoid cystic carcinoma).^[61]^

The aspects related to surgical technique are also found in the present study. The majority of malignant tumors were completely excised, while benign tumors were mostly treated with subtotal excision in order to preserve the facial nerve. For grade III or IV malignant tumors, one can choose to undergo postoperative chemotherapy along with radiotherapy or participate in clinical trials for innovative treatments.^[62]^

In the nonneoplastic lesions, the treatment is varied depending on each individual pathology. Thus, it can be an etiological 1 (for example smoking cessation), with local or systemic administration of corticosteroids, antibiotic therapy or antiviral medication, or lithotripsy in cases of sialolithiasis. Also, in certain cases, surgical resection can be opted for.^[5]^

The study’s limitations lie in its retrospective design and the availability of insufficient or incomplete data concerning the patients working and living conditions, as well as their lifestyle. What makes this study particularly novel is its inclusion of epidemiological data that considers all biological characteristics of salivary gland disorders. We have also discovered relevant elements, such as the consistent increase in frequency exclusively among benign tumors in females, alongside notable new findings such as an increase in malignant pathology in males and the dominance of certain lesions depending on the hemicranium location. These elements can be the basis of future research studies.

## 5. Conclusion

In conclusion, this study has emphasized the key epidemiological aspects of salivary gland pathology, alongside conducting a comprehensive review of the relevant literature. We observed an older age distribution in primary and secondary malignant cases. Although males are more commonly affected, we have observed statistically significant differences in the gender of the patients and the biological characteristics of the lesions. Also, a particular aspect consisted in the distribution of some lesions according to the topography of the hemicranium.

We have observed both similarities with the specialized literature and unique aspects that support the heterogeneity of this pathology, regarding the frequency of the affected salivary gland and the types of lesions. Therefore, the study is valuable for the development of medical services, making a significant impact on both research and public health.

## Author contributions

**Conceptualization:** Sorin Vamesu, Oana Andreea Ursica.

**Data curation:** Sorin Vamesu, Cristian Ionut Orasanu.

**Formal analysis:** Sorin Vamesu, Mariana Aschie, Georgeta Camelia Cozaru.

**Funding acquisition:** Sorin Vamesu.

**Investigation:** Oana Andreea Ursica, Ana Maria Gurita, Cristian Ionut Orasanu.

**Methodology:** Mariana Deacu, Mariana Aschie, Madalina Bosoteanu.

**Resources:** Sorin Vamesu, Raluca Ioana Voda, Mariana Deacu, Madalina Bosoteanu.

**Software:** Ana Maria Gurita, Raluca Ioana Voda.

**Supervision:** Sorin Vamesu, Cristian Ionut Orasanu.

**Validation:** Sorin Vamesu, Oana Andreea Ursica, Ana Maria Gurita, Raluca Ioana Voda, Cristian Ionut Orasanu.

**Visualization:** Oana Andreea Ursica, Ana Maria Gurita, Anca Florentina Mitroi.

**Writing – original draft:** Oana Andreea Ursica, Ana Maria Gurita, Raluca Ioana Voda, Cristian Ionut Orasanu.

**Writing – review & editing:** Sorin Vamesu, Mariana Deacu, Mariana Aschie, Madalina Bosoteanu.
